# Multiple Cutaneous Metastases as Initial Presentation in Advanced Colon Cancer

**DOI:** 10.1155/2018/8032905

**Published:** 2018-04-30

**Authors:** Sudheer Nambiar, Asha Karippot

**Affiliations:** ^1^Pulmonary Critical Care Medicine, Cancer Treatment Centers of America, Tulsa, OK, USA; ^2^Hematology and Oncology, Cancer Treatment Centers of America, Tulsa, OK, USA

## Abstract

Skin metastases from advanced colorectal cancer are relatively rare and occur most often when the cancer is advanced, following the spread to other organs. Cutaneous metastases occur in about 3% of advanced colorectal cancers. We present an extremely rare case of a 68-year-old woman with advanced ascending colon adenocarcinoma that presented with multiple rapidly progressing painless cutaneous metastatic lesions with no other distant metastases. Of all the tumors, breast cancer most commonly spreads as cutaneous metastasis is followed by lung, colorectal, renal, ovarian, and bladder cancers. Cutaneous metastases can present in a variety of clinical manifestations, such as a rapidly growing painless dermal or subcutaneous nodule with intact overlying epidermis or as ulcers. In cases where the cutaneous deposit is isolated, as in visceral metastasis, there is a role for radical management such as wide local excision and reconstruction. In our patient, since she had multiple cutaneous metastases she began treatment with palliative systemic combination chemotherapy.

## 1. Introduction

Cutaneous metastasis of colorectal adenocarcinoma is a rare event (2.3% to 6%) that usually occurs few years after the detection or resection of the primary tumor [1]. It seldom occurs before the identification of the primary tumor. When they present with cutaneous metastasis often there is involvement of secondary organs, such as the liver. Multiple cutaneous metastases as initial presentation with no visceral metastasis are a rare occurrence in colorectal cancer. Cutaneous metastases can present in a variety of clinical manifestations, such as a rapidly growing painless dermal or subcutaneous nodule with intact overlying epidermis or as ulcers.

## 2. Case Presentation

**Figure 1:**
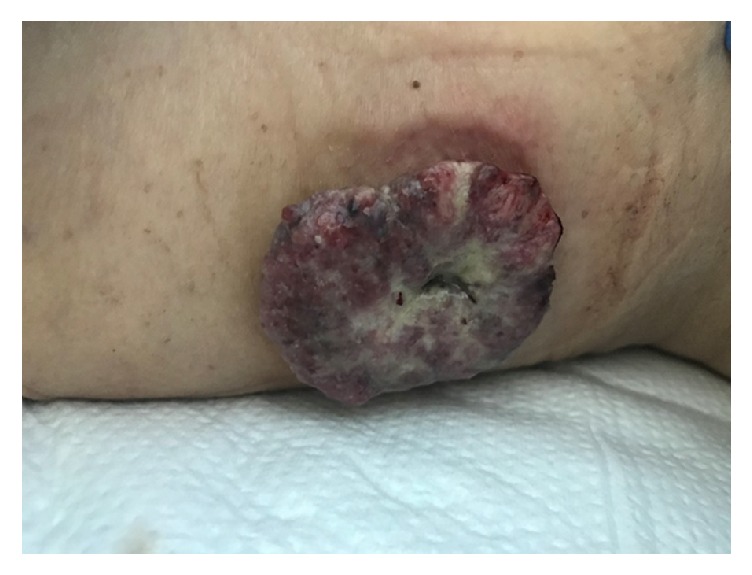
Cutaneous lesion in the lateral aspect of right thigh measuring 4 cm × 3 cm.

**Figure 2:**
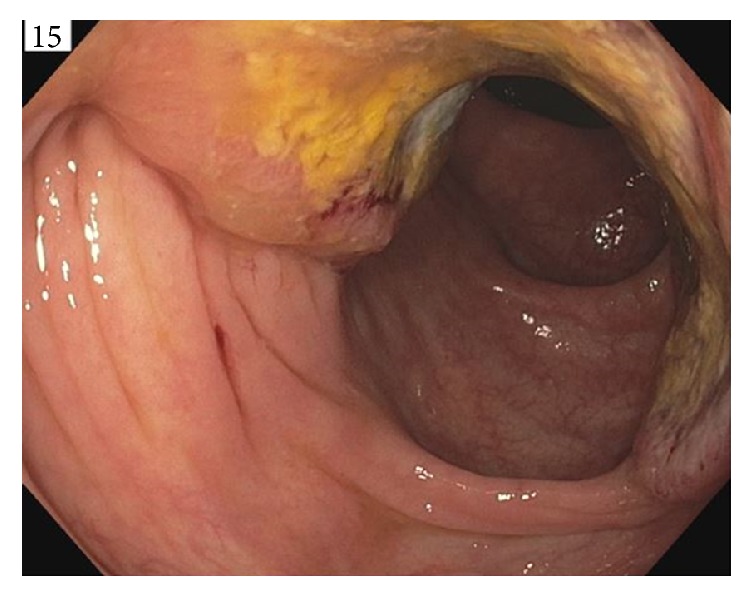
A large, friable, and infiltrative tumor which occupied 50 to 74% of the circumference of the ascending colon.

**Figure 3:**
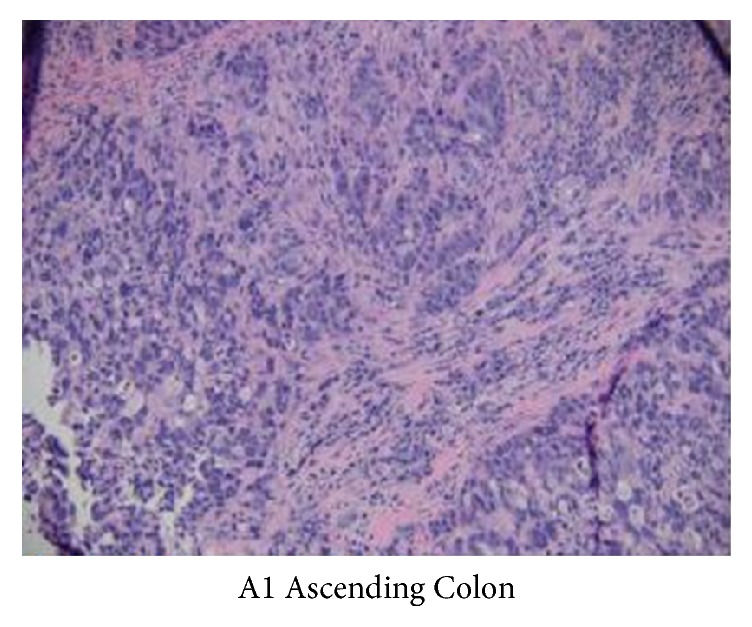
Ascending colon biopsy showing poorly differentiated invasive adenocarcinoma (H&E, ×400).

A 68-year-old female with no significant past medical history presented with a rapidly progressing painless extrinsic irregular mass on her lateral aspect of right thigh for three-month duration (Figure 1). She reported no trauma or previous surgery to that site. She noted occasional bleeding from this lesion. She denied weight loss, malena, constipation, hematuria, or abdominal pain. Her last colonoscopy was 10 years ago, the findings of the test were unremarkable. Her vital signs were stable. She had a biopsy of this cutaneous lesion by a local surgeon which revealed metastatic adenocarcinoma. An immune-peroxidase stain panel showed CAM5.2 positive, 34 BE12 positive, CDX-2 positive, CEA positive, CK-7 negative, CK-20 negative, and TTF-1 negative consistent with metastatic adenocarcinoma either bladder or gastrointestinal as primary. She presented herself to our institute for further care. Physical examination was only positive for a cutaneous lesion in the lateral aspect of her right thigh measuring 4 cm × 3 cm, a second lesion in the right upper posterior chest measuring 2 cm × 1.3 cm, and third lesion involving the right axilla measuring 1 cm × 1.5 cm. After initial evaluation by her medical oncologist she was referred to gastroenterologist for endoscopy. She had upper and lower endoscopy of the gastrointestinal tract. Upper endoscopy study was benign. Colonoscopy revealed multiple polyps in the transverse colon, descending colon, and rectum. Additionally, there was a large, friable, and infiltrative tumor which occupied 50 to 74% of the circumference of the ascending colon. It was not causing significant narrowing (Figure 2). The tumor bled on contact. Multiple cold forceps biopsies were taken. The specimens were collected for pathology. The tumor measured approximately 6 cm × 3 cm in size. Biopsy of right thigh mass was done. The cecum and rest of the colon appeared to be normal. An immune-peroxidase stain panel done showed CAM5.2 positive, 34 BE12 positive, CDX-2 positive, CEA positive, CK-7 negative, and CK-20 negative, TTF-1 negative consistent with adenocarcinoma of colon (Figure 3). A PET scan confirmed hypermetabolic activity only at ascending colon and in areas of cutaneous soft tissue masses. She was diagnosed to have stage 4 adenocarcinoma of the right colon with multiple cutaneous metastases and was started on combination chemotherapy with folinic acid, fluorouracil, and oxaliplatin (FOLFOX). Currently she is undergoing chemotherapy cycles.

## 3. Discussion

Cutaneous metastases of cancer are rare, occurring in about 1.3% of cases at the time of presentation of the primary tumor. Cutaneous metastases occur in about 3% of advanced colorectal cancers [1]. Mostly they present along with metastatic disease at other visceral sites [2] and mainstay of management is palliative. From the review of case reports, more than 60–65% of cases have active visceral metastasis at the time of finding cutaneous metastasis. However, infrequently it can be the first signal of malignancy, which occurs with greater frequency in breast and lung carcinoma, followed by kidney and ovarian cancer [3, 4]. Synchronous multiple cutaneous metastases as initial presentation in colon cancer excluding metastasis to visceral organs are an extremely rare occurrence in colorectal carcinoma as in our case report.

Of all tumors, breast cancer most commonly spreads as cutaneous metastasis by direct, hematogenic, and lymphatic pathways [3], with incidence of 24% according to a meta-analysis by Krathen et al. [4]. Lung, colorectal, renal, ovarian, and bladder cancer have similar rates of cutaneous metastases, which vary from 3.4% to 4%, mainly through blood and lymphatic dissemination. Cutaneous metastases often favor areas close to the primary malignancy, such as lung cancer and skin metastases on the trunk. The most frequent cutaneous site of colon cancer metastasis is the surgical scar in the abdomen following the removal of the primary malignancy. Several clinical appearances have been described in cutaneous metastases. This variable clinical morphology included ulcers, papules, nodules, plaques, and tumors. Histopathological patterns of cutaneous metastases involve the dermis, namely, nodular, infiltrative, diffuse, and intravascular [5]. Sister Mary Joseph nodule is well known entity where metastasis is noted in the umbilicus [6, 7]. Metastases to the thigh and back of the trunk are extremely rare as seen in our patient.

Early diagnosis will be the key element in its effective management. the American Society of Clinical Oncology (ASCO) guidance already recommends physical examination to rule out cutaneous deposits in colorectal cancers. Patient education to report any changes in skin is crucial in early detection and should be included in information leaflets and guidance for surveillance. Immunohistochemical screening methods assist in diagnosing the primary tumors of origin [8]. Such cutaneous metastasis should trigger a search for any visceral metastasis, the best modality available being PET scan, which is also the only other way of detecting other skin lesions unless clinically obvious. In cases where the cutaneous deposit is isolated, as in visceral metastasis, there is a role for radical management such as wide local excision and reconstruction should be considered. In our case the patient had multiple cutaneous metastases and so was treated with systemic palliative chemotherapy. Survival varies from one to thirty-four months following the diagnosis of cutaneous metastasis. The average survival of patients after the diagnosis of cutaneous metastasis of colon carcinoma is 18 months [7]. There is no median survival rate information available from the literature regarding colorectal cancers presenting as multiple cutaneous metastasis alone. Identification of cutaneous metastasis from an internal malignancy indicates poor prognosis [9, 10]. However, the morbidity related to such metastasis can be reduced by early detection, which requires awareness and meticulous physical examination on the part of the patient and the physician.

## Data Availability

Patient data are obtained by reviewing the medical records.
